# Neurotrophic basis to the pathogenesis of depression and phytotherapy

**DOI:** 10.3389/fphar.2023.1182666

**Published:** 2023-04-06

**Authors:** Huiqin Wang, Yantao Yang, Gang Pei, Zhenzhen Wang, Naihong Chen

**Affiliations:** ^1^ Hunan University of Chinese Medicine and Hunan Engineering Technology Center of Standardization and Function of Chinese Herbal Decoction Pieces, Changsha, Hunan, China; ^2^ State Key Laboratory of Bioactive Substances and Functions of Natural Medicines, Institute of Materia Medica and Neuroscience Center, Chinese Academy of Medical Sciences and Peking Union Medical College, Beijing, China

**Keywords:** depression, neurotrophic factors, pathogenesis, neurotrophic basis, phytotherapy, phytochemicals, antidepressant

## Abstract

Depression is a major neuropsychiatric disease that considerably impacts individuals’ psychosocial function and life quality. Neurotrophic factors are now connected to the pathogenesis of depression, while the definitive neurotrophic basis remains elusive. Besides, phytotherapy is alternative to conventional antidepressants that may minimize undesirable adverse reactions. Thus, further research into the interaction between neurotrophic factors and depression and phytochemicals that repair neurotrophic factors deficit is highly required. This review highlighted the implication of neurotrophic factors in depression, with a focus on the brain-derived neurotrophic factor (BDNF), glial cell line-derived neurotrophic factor (GDNF), vascular endothelial growth factor (VEGF), and nerve growth factor (NGF), and detailed the antidepressant activities of various phytochemicals targeting neurotrophic factors. Additionally, we presented future opportunities for novel diagnostic and therapeutic strategies for depression and provided solutions to challenges in this area to accelerate the clinical translation of neurotrophic factors for the treatment of depression.

## 1 Introduction

Depression is one of the most common and serious neuropsychiatric disorders, affecting people’s thoughts, behaviors, interests, and feelings. Clinical patients with depression are characterized by several manifestations such as gloomy mood, loss of interest, sleep disturbances, etc. (Malhi and Mann, 2018; Wang et al., 2021a). While the pathogenesis of depression is multifactorial and poorly understood. Its diverse manifestations, erratic course and prognosis, and inconsistent responsiveness to therapy pose a challenge to its detection, diagnosis, and management (Leung et al., 2022). Therefore, it is necessary to investigate theoretical underpinnings and novel targets for early prevention and accurate diagnosis of depression. Additionally, conventional antidepressants display remarkable limitations, such as the delayed onset of action, low response rates, and relapse following medication discontinuation, impeding treatment compliance in patients with depression (Sabella, 2018). Accordingly, identifying non-adverse and side-effect-free alternatives to traditional antidepressants is vital to improving drug adherence in depressed individuals.

## 2 Neurotrophic basis of depression

**FIGURE 1:**
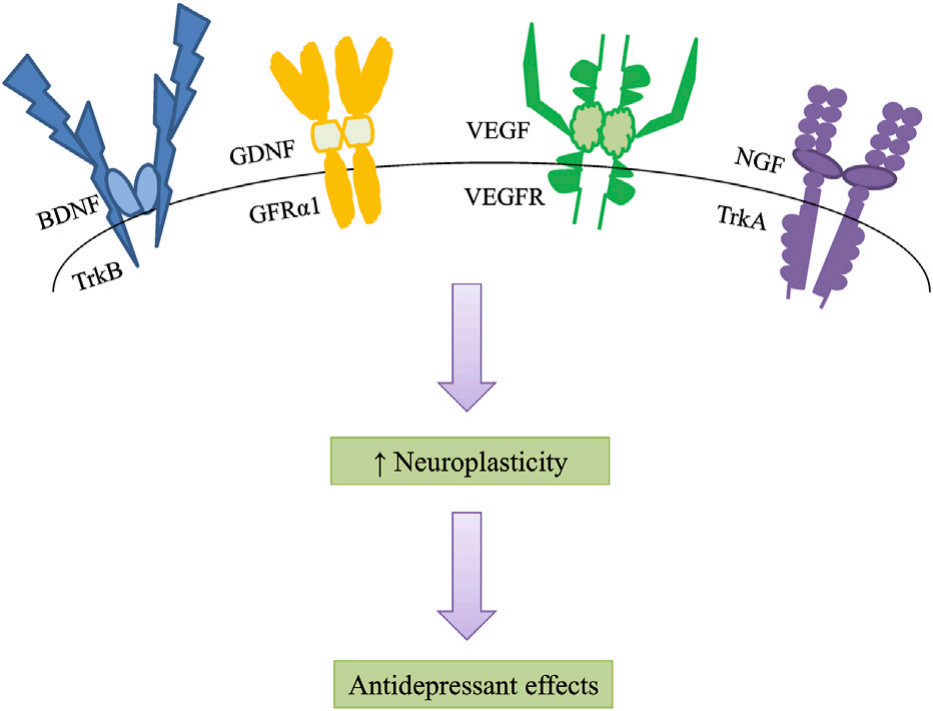
Neurotrophic factors increase neuroplasticity, especially synaptic plasticity, neurotransmission, and neuronal survival, growth, and differentiation. An increase in neuroplasticity is likely to induce antidepressant effects. BDNF, brain-derived neurotrophic factor; TrkB, tropomyosin-related kinase receptor B; GDNF, glial cell line-derived neurotrophic factor; GFRα1, GDNF-family receptor-α1; VEGF, vascular endothelial growth factor; VEGFR, vascular endothelial growth factor receptor; NGF, nerve growth factor; TrkA, tropomyosin-related kinase receptor A.

Neuroplasticity is responsible for neurogenesis and the modification of mature neuronal morphology (Allen and Lyons, 2018). Limiting neurogenesis prevents antidepressant action and has been substantiated to depression-like syndromes, especially under stressful situations (Castreń, 2013). Therefore, neurogenesis has been proposed to facilitate stress resilience, which might be the foundation of antidepressant therapeutic benefits. Neurotrophic factors are essential mediators of neuroplasticity among several candidates (Song et al., 2017), to boost neuroplasticity, particularly synaptic plasticity, neurotransmission, and neuronal survival, growth, and differentiation (Thoenen, 1995; Wang et al., 2022). Furthermore, the increase in neuroplasticity is expected to attract antidepressant benefits (Figure 1). The secretion of neurotrophic production increased after antidepressant treatment, promoting the survival of neurons and shielding them from stress-related damage. As a result, the onset of depression is implicated in the impairment of neurotrophic factor signaling (Table 1). Although efforts have been made to understand the neurotrophic basis of the pathogenesis of depression, many fundamental questions regarding their mechanisms of action remain to be addressed systematically to better understand the complicated neurotrophic basis in depression treatment.

**TABLE 1:** Relationship between neurotrophins and the pathogenesis of depression.

Neurotrophins	First discovered	Changes in MDD	References
BDNF	1980s	↓Amygdala	Hofer and Barde, (1988), Leibrock et al., 1989, Guilloux et al., 2012, Wook Koo et al., 2016, Zhang, (2011)
	Barde	↓Plasma	
		↓Serum	
		↓DG	
		↑NAc	
GDNF	1993	↓Serum	Lin et al., 1993, Zhang et al., 2009
	Lin		
VEGF	1989	↓Plasma	Leung et al., 1989, Isung et al., 2012, Castillo et al., 2020, Lee and Kim, (2012)
	Ferrara	↑Plasma	
NGF	1956	↓Serum	Levi-Montalcini and Angeletti, (1968), Wiener et al., 2015
	Levi-Montalcini		

MDD, major depressive disorder; BDNF, brain-derived neurotrophic factor; GDNF, glial cell-derived neurotrophic factor; VEGF, vascular endothelial growth factor; NGF, nerve growth factor; ↓, decrease; ↑, increase; DG, dentate gyrus; NAc, nucleus accumbens.

### 2.1 Brain-derived neurotrophic factor and depression

Brain-derived neurotrophic factor (BDNF), an essential member of the neurotrophic factor family, was initially discovered in the brain of a pig by Barde in the 1980s (Leibrock et al., 1989). BDNF, primarily synthesized in neurons, is ubiquitously distributed throughout the central nervous system (CNS). It is involved in the repair of synaptic plasticity, the transduction of 5-hydroxytryptamine (5-HT) signaling, and the level of 5-HT in the brain (Bhattarai et al., 2020; Costa et al., 2022). Consistent reports have certified that BDNF is associated with the occurrence, development, and management of depression, and it has received the most attention in the neurobiology of depression among any neurotrophic factors.

Researchers are constantly investigating the relationship between variations in activity and content of BDNF and the occurrence or outcome of depression. BDNF deficiency in the amygdala is visible in women with major depressive disorder (MDD) (Guilloux et al., 2012). *Postmortem* analysis revealed that plasma BDNF levels are lower in depressed patients than that in controls (Gadad et al., 2021). Moreover, a series of experiments have confirmed that intracerebral administration of BDNF has antidepressant efficacy in depressive animal models (Deltheil et al., 2009). Antidepressant studies targeting BDNF have the potential to be one of the most valid strategies for the development of novel antidepressant medications. Subsequently, Fukumoto et al. demonstrated that the antidepressant effect of (2R, 6R)-Hydroxynorketamine [(2R, 6R)-HNK], a ketamine metabolite that can produce rapid and sustained antidepressant actions in animal models without side effects of ketamine, was mediated through active-dependent release of BDNF in the medial prefrontal cortex (mPFC), sufficiently demonstrating the indispensable role of BDNF in antidepressant treatment (Fukumoto et al., 2019).

However, the association between BDNF and depression has not yielded conclusive results. Tropomyosin-related kinase B (TrkB), a specific BDNF receptor, has been pointed to activate BDNF-TrkB signaling to exert the antidepressant action (Rantamäki et al., 2007), and ketamine improves postoperative depression symptoms by upregulating BDNF-TrkB signaling as well. However, Wook Koo et al. pointed out that chronic social defeat stress increased BDNF expression level in the nucleus accumbens, and local knockout of the *BDNF* gene in the ventral tegmental area reduced depression-like phenotypes, demonstrating that BDNF signaling induces depression susceptibility (Wook Koo et al., 2016). The role of BDNF acts variably in diverse brain regions, warranting additional study of individual mechanisms. Besides, a substantial reduction in BDNF levels in rheumatoid arthritis patients with depression were detected (Cheon et al., 2018; Nerurkar et al., 2019), and the severity of depression is related to fatigue, poor BDNF expression, and serious state of rheumatoid arthritis. Therefore, BDNF levels might be potential biomarkers for the prediction or monitoring of depression.

Much work on BDNF has recently been reported in this field, while the following issues should be highlighted: there are differences in the stability of BDNF levels measured by different laboratories in whole blood, serum, and plasma (Karege et al., 2005; Suwalska et al., 2010; Arosio et al., 2021), which may be attributed to differences in enzyme-linked immunosorbent assay methods or sampling tubes; the discrepant level and mechanism of BDNF in various brain regions are different, which deserves further study; the more stable and accurate BDNF measurements should be determined and find out which source of BDNF is the most reliable biomarker of MDD, as concentrations of BDNF markers in the circulation do not always reflect the CNS concentrations.

### 2.2 Glial cell line-derived neurotrophic factor and depression

Glial cell line-derived neurotrophic factor (GDNF) is a neurotrophic factor of the *β* family that is widely distributed throughout the brain and regulates the noradrenergic and GABAergic systems. It was first purified and named in 1993 by Lin et al. (Lin et al., 1993). GDNF is one of the most efficient neurotrophins, influencing the growth, survival, and activity of midbrain dopaminergic neurons, protecting neurons from oxidative stress, and constituting major players in the development and function of hippocampal neurons (Yang et al., 2001; Bonafina et al., 2019).

A *postmortem* study on characters with MDD found that the level of GDNF decreased in PFC and the concentration of GDNF in the amygdala reduced as well (Michel et al., 2008; Järvelä et al., 2011; Tang et al., 2023), implying that lower serum GDNF may be involved in the pathophysiology of MDD. Zhang et al. investigated whether the serum GDNF of patients with MDD differed from that of the healthy control group before antidepressant treatment and whether it could affect serum GDNF expression in patients with MDD after antidepressant treatment (Zhang et al., 2010). The results revealed that serum GDNF levels were conspicuously lower in MDD patients before treatment than that in healthy volunteers. Antidepressants could increase *GDNF* mRNA and protein levels, suggesting the increased GDNF might contribute to the improvement of depression (Maheu et al., 2015). Furthermore, central GDNF signaling may also be a potential antidepressant target. High plasma GDNF levels may be implicated in the pathophysiology of late-onset depression and cognitive impairment in late-onset depression patients (Wang et al., 2011). Consequently, a reduction in GDNF levels might be a biomarker of depressed status.

Based on the above studies on the interaction between GDNF and depression, researchers can recognize that: whether the influence of peripheral and central GDNF on the pathogenesis of depression is not completely clear; supplementation of exogenous GDNF has an antidepressant effect. When it comes to exogenous GDNF supplied to serum, plasma, and whole blood, the optimal strategy must be determined.

### 2.3 Vascular endothelial growth factor and depression

Vascular endothelial growth factor (VEGF) is an effective mitogen and survival factor for endothelial cells and neurons, as well as a modulator of synaptic transmission (Vargish et al., 2017). In 1989, Ferrara et al. isolated and cloned this substance and named it (Leung et al., 1989). In addition to angiogenic action (Apte et al., 2019), current research has revealed the neurotrophic and neuroprotective potentiality of VEGF in the CNS (Jin et al., 2002; Sene et al., 2015). For example, VEGF influences the pathophysiology of hippocampal neurogenesis and depression, contributes to the occurrence of hippocampal neurons, and shields stress-related neurons from damage (Cao et al., 2004; Kirby et al., 2015), which is essential for antidepressant therapy. Inhibiting the expression of VEGF receptor 2 in nerve cells impairs hippocampal-dependent synaptic plasticity and emotional memory consolidation (De Rossi et al., 2016).

Current clinical research on the correlation between VEGF and the onset of depression has not yielded consistent results. When compared to that in healthy volunteers, the expression of VEGF in patients with depression tends to increase in serum and plasma (Castillo et al., 2020), while quite a few studies have detected an average decrease in VEGF levels in patients with depression (Du Preez et al., 2021), which may be due to inadequate assessment of environmental factors such as gender, age, and body mass index. VEGF can predict the response of antidepressant treatment, suggesting that it is a possible biomarker and mediator engaged in neuroplastic processes (Castillo et al., 2020).

The findings make an important contribution to this expanding field of VEGF research, which can be emphasized as follows: even though BDNF is currently the most studied neurotrophic factor in neurobiology in MDD, the effects of VEGF on the pathogenesis of depression should not be underestimated, which means that the relationship between VEGF and depression should be investigated thoroughly; the correlation between VEGF and depression remains inconsistent, so the effect of VEGF on depression should be designed to combine with environmental variables.

### 2.4 Nerve growth factor and depression

Nerve growth factor (NGF), an essential member of the neurotrophic factor family, was first isolated in 1956 by Levi-Montalcini (Levi-Montalcini and Angeletti, 1968). It is primarily generated in the cortex, hippocampus, and hypothalamus, but it is also found in the peripheral nervous system and the immune system (Meng et al., 2022). NGF has a strong affinity for TrkA (Riccio et al., 1997; Deppmann et al., 2008). Owing to its participation in neuroplasticity, learning, and memory, NGF is essential for the response to stress and the regulation of the neuro-endocrine-immunity system (Mohammadi et al., 2018).

NGF plays an important role in the pathogenesis of depressive symptoms and the response to antidepressant treatment, which can be seen from that exogenous NGF could induce antidepressant-like effects in rodent depression models (Mezhlumyan et al., 2022). In a study examining the effects of NGF on depression, NGF improved depression-like behaviors like fluoxetine and amitriptyline (McGeary et al., 2011), suggesting NGF is involved in the pathogenesis of depressive symptoms and the response to antidepressant treatment. To test whether NGF is associated with the etiology of depression or suicide risk, Wiener et al. examined changes in serum NGF levels in MDD patients with or without suicidal risk (Wiener et al., 2015). The results showed that the serum levels of NGF in the MDD group and MDD along with suicide risk group were significantly reduced, however, there was no difference between the MDD group and MDD along with suicide risk group, from where we could point out that NGF was a biomarker of MDD. It may be associated with the diagnosis of MDD but not with the severity of symptoms. Early adverse experiences in humans, for instance, maternal deprivation, are linked to an increased risk of mental illnesses such as anxiety and MDD, and data from Cirulli et al. showed that NGF was a potential candidate for adverse events in brain dysfunction and a neuroendocrine marker for the different responses of male and female rhesus monkeys suffering from maternal deprivation (Cirulli et al., 2009).

Based on the above NGF and depression studies, researchers can find that the presence of suicide risk does not affect the serum levels of NGF, suggesting NGF may be associated with the diagnosis of MDD but not with the severity of symptoms.

## 3 Phytotherapy on depression targeting neurotrophic factors

Despite the fact that conventional antidepressant therapy can help relieve symptoms of depression, concerns have been raised regarding complementary therapies due to the drawbacks of the current medications. Phytochemical constituents, a ubiquitous class of plant secondary metabolites, have revealed their therapeutic benefits in many indications, including mental disorders (Raimundo et al., 2022). The use of phytochemicals is a complementary method to conventional antidepressants to provide therapeutic advantages and avoid unwanted adverse reactions. To date, subsequent evidence indicates that impairment in neurotrophic basis is associated with depression, and phytochemicals targeting neurotrophic factors exert antidepressant properties. It is thus not surprising that the focus of the pharmacological study on phytochemicals for the treatment of depression has been targeting neurotrophic factors, among which BDNF, GDNF, VEGF, and NGF are the most relevant neurotrophins. For example, curcumin, one of the few phytochemicals that have found its way into human studies, exerts antidepressant effects by improving the levels of hippocampal BDNF (Sanmukhani et al., 2014; Fusar-Poli et al., 2020). Besides, resveratrol is a natural polyphenol that could improve the reduction in sucrose preference in rats by promoting BDNF and GDNF levels (Liu et al., 2014; Couteur et al., 2021). GDNF and NGF could be inducted by olive polyphenol administration in the hippocampus and olfactory bulbs of mice (De Nicoló et al., 2013). Naringin increased the expression of BDNF and VEGF in rat models (Rong et al., 2012; Viswanatha et al., 2022). Table 2 manifested other phytochemicals targeting neurotrophic factors for depression treatment.

**TABLE 2:** Antidepressant effects induced by phytochemicals based on neurotrophic factors.

Phytochemicals	Behavioral effects	Neurotrophic mechanisms	References
Auraptene	↓Immobility time in FST and TST	↑*GDNF* mRNA	Amini-Khoei et al., 2022, Furukawa et al., 2020
Baicalein	↓Immobility time in FST and TST	↑BDNF/TrkB/CREB pathway	Zhao et al., 2021, Liu et al., 2022
	↑Sucrose preference in SPT	↓Inflammatory cytokines	
	↑OFT	↑BDNF	
Catalpol	↓Immobility time in FST	↓NLRP3 inflammasome and neuroinflammation	Wang et al., 2021d, Wang et al., 2015, Xu et al., 2010
	↑OFT	↑BDNF expression and TrkB, ↓COX-2 expression and PGE2	
		↑GDNF	
Chrysin	↓Immobility time in FST	↑BDNF	Filho et al., 2016, Ma et al., 2020
		↓Cytokines and 5-HT	
	↑OFT	↓Ca^2+^ availability	
		↑cAMP/PKA and NO/cGMP signaling pathways	
Curcumin	↓Immobility time in FST	↑Hippocampal synaptic plasticity	Fan et al. (2021)
	↑Sucrose preference in SPT	↑Hippocampal BDNF	
Dimethyl fumarate	↓Immobility time in FST	↑Hippocampal BDNF and *β*-catenin	Abd El-Fattah et al. (2018)
	↑Sucrose preference in SPT		
	↑Sucrose preference in SPT		
Emodin	↓Immobility time in FST and TST	↑BDNF	Ahn et al., 2016, Zhang et al., 2021b
	↑Sucrose intake in SPT	↓Inflammatory responses	
Eugenol	↓Immobility time in FST	↑BDNF	Irie et al. (2004), Norte et al., 2005
Genipin	↓Immobility time in FST and TST	↑Hippocampal BDNF	Ye et al. (2018)
Ginsenosides Rb1	↑social interaction	↑BDNF	Jiang et al., 2022, Zhang et al., 2021a, Liang et al., 2010
	↓Immobility time in FST and TST	↑NGF	
Ginsenoside Rg1	↓Immobility time in FST and TST	↑Hippocampal BDNF	Wang et al., 2021c, Jiang et al., 2012, Liang et al., 2010, Wang et al., 2021b
	↑Sucrose preference in SPT	↑NGF	
	↑OFT	↑Cx43-based gap junction	
Hesperidin	↑Sucrose preference in SPT	↑BDNF	Sharma et al., 2021, Li et al., 2016, Zhu et al., 2020
	↓Immobility time in FST and TST	↑GDNF	
Hyperforin	↓Immobility time in TST	↑BDNF	Pochwat et al. (2018)
Hypericin	↑Sucrose preference in SPT	↓PI3K/Akt pathway	Zhai et al., 2015, Zhang et al., 2015, Thong et al., 2006, Lavie et al., 2005
	↑Body weight	↑VEGF	
	↓Immobility time in TST	↓Phosphorylation of ERK1/2	
Macranthol	↑Sucrose preference in SPT	↑BDNF	Luo et al., 2015, Weng et al., 2019
Magnolol	↑Sucrose preference in SPT	↓M1 polarization	Tao et al. (2021)
Naringenin	↑Sucrose preference in SPT	↑BDNF	Bansal et al., 2018, Eraky et al., 2023
		↓Inflammatory cytokines	
Naringin	↓Immobility time in FST and TST	↑BDNF/TrkB/CREB pathway	Gao et al., 2022, Rong et al., 2012, Viswanatha et al., 2022
		↑VEGF	
Oleanolic acid	↓Immobility time in FST and TST	↑Hippocampal BDNF	Fajemiroye et al. (2014)
Olive polyphenol	_	↑GDNF and NGF in the hippocampus and olfactory bulbs	De Nicoló et al. (2013)
Orcinol glucoside	↓Immobility time in FST and TST	↑BDNF/TrkB/CREB pathway	Li et al. (2021)
	↑Sucrose preference in SPT		
	↑OFT		
Paeoniflorin	↑Sucrose preference in SPT	↑ERK1/2 pathway	Tang et al., 2021, Tian et al., 2021
	↑function of balance control and motor coordination in the BBT	↓Pyroptosis CASP-11/GSDMD pathway	
Piperine	↑Sucrose preference in SPT	↑BDNF	Ren and Zuo, (2019), Mao et al., 2014, Wattanathorn et al., 2008
	↑Spontaneous locomotor behavior		
	↓Immobility time in FST		
Quercetin	↑Bodyweight gain	↑BDNF in both the hippocampus and PFC	Ke et al. (2020)
	↑Saccharin preference index		
	↓Immobility time in FST		
Resveratrol	↑Sucrose preference in SPT	↑Peroxisome proliferator–activated receptor-γ coactivator	Abd El-Fattah et al., 2018, Smeding et al., 2012
		1α abundance and function	
		BDNF in both the hippocampus and PFC	
Tetrandrine	↓Immobility time in TST and FST	↑BDNF	Gao et al. (2013)

FST, forced swimming test; TST, tail suspension test; SPT, sucrose preference test; OFT, open field test; BBT, beam balance test; GDNF, glial cell-derived neurotrophic factor; BDNF, brain-derived neurotrophic factor; VEGF, vascular endothelial growth factor; NGF, nerve growth factor; TrkB, tyrosine kinase receptor B; cAMP, cyclic adenosine monophosphate; CREB, cAMP-response element binding protein; NLRP3, NOD-like receptor thermal protein domain associated protein 3; COX-2, cyclooxygenase-2; PGE2, prostaglandin E2; GDNF, PKA, protein kinase A; CASP-11, caspase-11; GSDMD, pore-forming protein gasdermin D; PFC, prefrontal cortex.

In conclusion, it suggests that antidepressant-like effects of phytochemicals may be mediated, at least in part, by enhanced neurotrophic factors produced in the brain. Phytochemicals targeting neurotrophic factors are the potential to be profoundly developed and used in the future. Research into the biochemical and pharmacological effects of these bioactive constituents may uncover novel treatments for psychiatric illness or yield fresh insights into basic disease mechanisms.

## 4 Conclusion and perspectives

Depression is one of the most serious health challenges that affect the quality and duration of life substantially and disastrously. In terms of the therapeutic efficacy of depression, the limitations of traditional antidepressants remain notable. For example, a significant portion of patients with depression is prone to recurrence or unresponsive to various antidepressants (Daly et al., 2019). Additionally, it delays several weeks for 5-HT reuptake inhibitors, the mainstream antidepressants, to take action. Nevertheless, innovative therapeutics are still rare, owing in part to the difficulty of uncovering the underlying biological mechanisms of depression. As a result, the development of identifying novel therapeutic targets for depression is urgently required.

The expression and levels of BDNF, GDNF, VEGF, and NGF appear to be differentially altered in MDD patients compared to healthy persons, indicating that these molecules may constitute crucial roles in the pathophysiology of depression and antidepressant activity of treatment interventions. Coupled with new insights into the underlying mechanisms of depression, the rich abundance of chemical entities derived from herbs is proving to be an enticing resource in the search for effective therapy. Phytotherapy with a long history of useful applications is gaining popularity in pharmaceutical research. The active ingredients operating on multiple neurotrophic factors have been identified and extensively evaluated for therapeutic efficacies. Phytochemical components are more broadly available, tolerable, and presumably possess fewer negative effects in comparison to synthetic pharmaceutical medications, making them especially appealing for further exploitation and characterization for potential application in depression. Although animal research has yielded a plethora of candidates for phytotherapy, only a limited number of these compounds have made it into clinical trials. It is necessary to perform clinical trials to establish the therapeutic potential and validate the efficacy and safety of natural antidepressants.
